# *Vibrio cholerae* O1 and *Escherichia coli* O157:H7 from drinking water and wastewater in Addis Ababa, Ethiopia

**DOI:** 10.1186/s12866-024-03302-8

**Published:** 2024-06-20

**Authors:** Helina Mogessie, Mengistu Legesse, Aklilu Feleke Hailu, Tilahun Teklehaymanot, Haile Alemayehu, Rajiha Abubeker, Mogessie Ashenafi

**Affiliations:** 1 Microbiology Research Unit, Aklilu Lemma Institute of Pathobiology, Addis Ababa University, Addis Ababa, Ethiopia; 2 Ethiopian Public Health Institute, Bacteriology Directorate, Addis Ababa, Ethiopia; 3 Center for Food Security Studies, College of Development Studies, Addis Ababa University, Addis Ababa, Ethiopia

**Keywords:** Wastewater, Drinking water, *V. Cholerae* O1, *E. Coli* O157:H7, Multiple antibiotic resistance

## Abstract

**Background:**

In Addis Ababa, Ethiopia, open ditches along innner roads in residential areas serve to convey domestic wastewater and rainwater away from residences. Contamination of drinking water by wastewater through faulty distribution lines could expose households to waterborne illnesses. This prompted the study to assess the microbiological safety of wastewater and drinking water in Addis Ababa, identify the pathogens therein, and determine their antibiotic resistance patterns.

**Results Vibrio cholerae:**

O1, mainly Hikojima serotype, was isolated from 23 wastewater and 16 drinking water samples. Similarly, 19 wastewater and 10 drinking water samples yielded *Escherichia coli* O157:H7. *V. cholerae* O1 were 100% resistant to the penicillins (Amoxacillin and Ampicillin), and 51–82% were resistant to the cephalosporins. About 44% of the *V. cholerae* O1 isolates in this study were Extended Spectrum Beta-Lactamase (ESBL) producers. Moreover, 26% were resistant to Meropenem. Peperacillin/Tazobactam was the only effective β-lactam antibiotic against *V. cholerae* O1. *V. cholerae* O1 isolates showed 37 different patterns of multiple resistance ranging from a minimum of three to a maximum of ten antimicrobials. Of the *E. coli* O157:H7 isolates, 71% were ESBL producers. About 96% were resistant to Ampicillin. Amikacin and Gentamicin were very effective against *E. coli* O157:H7 isolates. The isolates from wastewater and drinking water showed multiple antibiotic resistance against three to eight antibiotic drugs.

**Conclusions:**

Open ditches for wastewater conveyance along innner roads in residence areas and underground faulty municipal water distribution lines could be possible sources for *V. cholerae* O1 and *E. coli* O157:H7 infections to surrounding households and for dissemination of multiple drug resistance in humans and, potentially, the environment.

**Supplementary Information:**

The online version contains supplementary material available at 10.1186/s12866-024-03302-8.

## Background

Water is the dominant component of living organisms and consumption of water is a basic requirement for survival. Access to safe drinking water is essential for human health and well being. Therefore, drinking water must be free from disease-causing organisms and poisonous chemicals. One of the Sustainable Development Goals, ‘Goal Six’, aims at ensuring availability and sustainable management of water and sanitation for all by 2030” [1]. To achieve the goal, water supply systems should be constructed or improved so that safe piped water at point-of-use is provided to consumers. Thus, a safe sanitation system should be designed and used to separate human excreta from human contact at all steps of the sanitation service chain [2].

In Addis Ababa and other cities in Ethiopia, households secure drinking water from treated municipality lines. Drinking water, however, is not sterile and low levels of microorganisms may persist in the treated water [3]. However, there were reports that drinking water at point-of-use was more contaminated than at the source in many developing countries, including Ethiopia [4–7]. Even worse, the prevalence of diarrheagenic bacteria was more frequent in water at point-of-use than in the public domain source water in a low-income community [8]. These findings are thus indicative of faulty drinking water distribution lines.

In Addis Ababa, it is common to see open ditches, serving as sewers, along innner roads in residential areas, to convey wastewater and rainwater away from residences. Open-ditch sewers are often blocked throughout the distribution line, and result in stagnant wastewater close to residences. This would eventually sink into the soil surrounding old, and possibly corroded and perforated, underground water distribution lines. It is reported that microorganisms in the surrounding could be sucked into drinking water distribution system by negative pressure [9], causing contaminant to pass into water distribution system as proved experimentally by Fontanazza et al. [10]. According to a study in Arbaminch, Ethiopa, faulty distribution lines could lead to the infiltration of bacterial contaminants into drinking water [5].

Several authors have isolated various pathogens from drinking water. A review by Kristanti et al. [11]. showed that pathogenic bacteria, viruses, protozoan parasites, and parasitic worms were isolated from drinking water from different parts of the world.

Isolation of *Vibrio cholerae* (*V. cholerae*) from environmental samples such as drinking water or sewer systems were reported from Nepal [12], Bangladesh [13], Uganda [14, 15] and Azerbaijan [16]. *Vibrio cholerae* causes cholera, manifested as life-threatening voluminous and watery diarrhea and vomiting. Outbreaks of cholera in different sub-cities of Addis Ababa have been reported at different times [17–20]. Although the pathogen was isolated from stool specimen, none of the studies, however, isolated *V. cholerae* from environmental samples, except one which reported the isolation of the pathogen from two holy water samples consumed [20].

Similarly, it has been long reported that *Escherichia coli* (*E. coli)* O157:H7 has been isolated from drinking water samples in different countries [21–23]. *E. coli* O157:H7 typically causes acute bloody diarrhea, which may lead to hemolytic-uremic syndrome. In Ethiopia, several reports indicate the isolation of *E. coli* O157:H7 only from meat and milk from cattle [24–27].

Various studies reported the antimicrobial sensitivity patterns of *V. cholerae* [28–30] or *E. coli* O157:H7 [31, 32]. Multiple drug resistance was detected in a considerable proportion of isolates belonging to both species [33].

The aims of this study were, therefore, (a) to isolate *V. cholerae* and *E. coli* O157:H7 from drinking water at point-of-use and wastewater in open ditches found along innner roads in residential areas in Addis Ketema and Akaki/Kality sub-cities in Addis Ababa, Ethiopia, and (b) to determine the antimicrobial resistance levels of the isolates (c) to detect Extended Spectrum Beta-Lactamase (ESBL) producing and Carbapenem resistant *V. cholerae* and *E. coli* O157:H7 .

## Materials and methods

### Study area and study period

A Cross sectional study was conducted in three woredas each of Addis Ketema and Akaki/Kality sub-cities to isolate *V. cholerae* and *E. coli* O157:H7 from drinking water and wastewater samples. Sample size was determined using simple population proportion formula.

$$\begin{gathered}n = \frac{{{{(Z\alpha /2)}^2}pq}}{{{d^2}}} \hfill \\\,\,\,\, = \frac{{{{(1.96)}^2} \times (0.14 \times 0.86)}}{{{{0.05}^2}}} = 185 \hfill \\ \end{gathered}$$

Where: *n* = sample size; Zα = risk expresses in z-score; p = expected prevalence (14%, based on 2019 Cholera outbreak); q = 1-p; d = absolute precision.

Considering a 10% non-responsive rate, the final sample size was taken as 206.

**Fig. 1:**
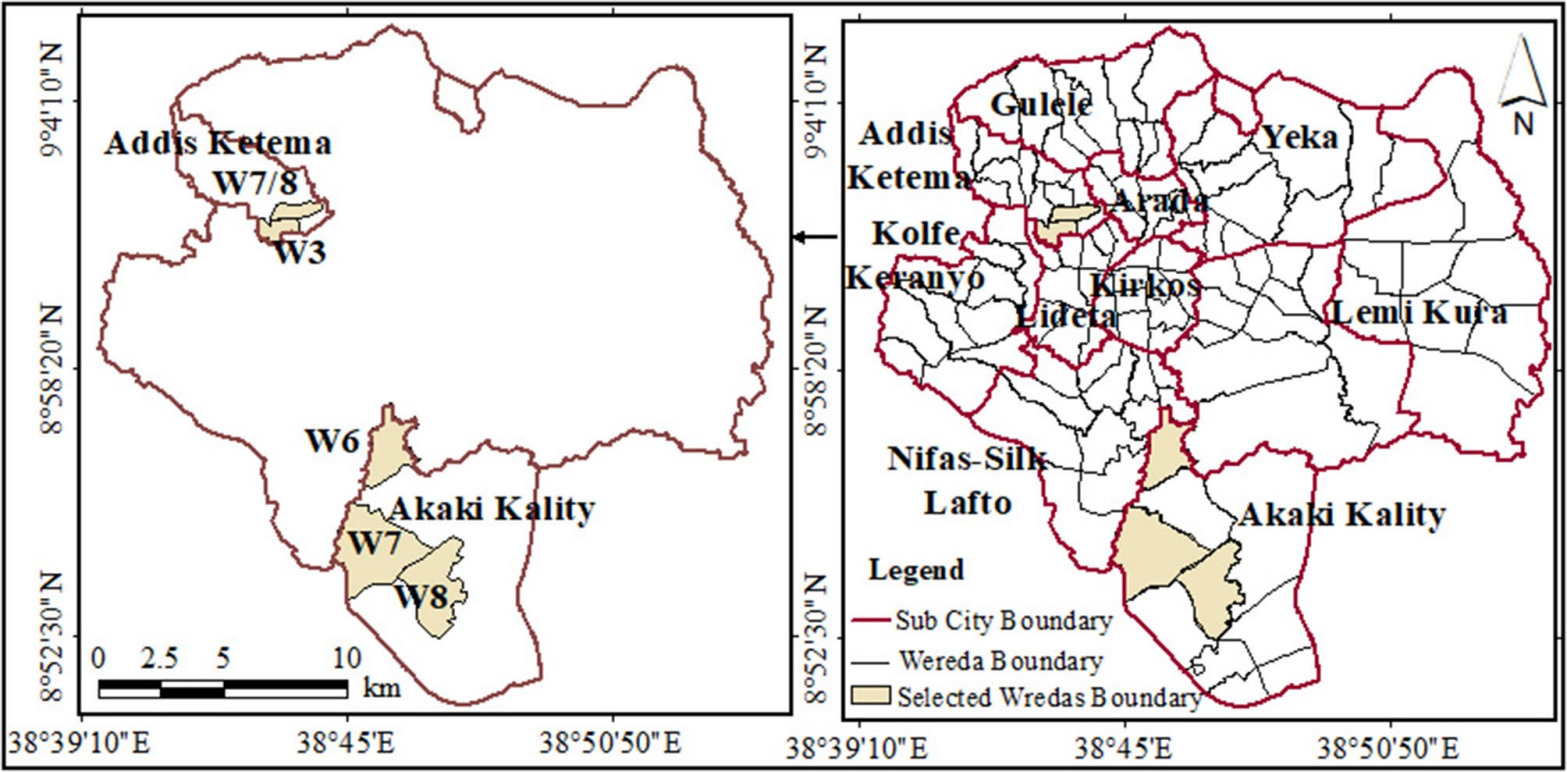
Map showing the study woredas in Addis Ketema and Akaki/Kality sub-cities, Addis Ababa

A total of 206 environmental water samples were aseptically collected from drinking water at point-of-use and surface sewerage from May to July 2023. These samples were collected from six different woredas within two sub cities in Addis Ababa (Fig. 1). A woreda is the smallest administrative unit in Addis Ababa. The sub cities were Addis Ketema sub city and Akaki Kality sub city. Three woredas each were selected from the two sub-cities: Woreda Three, Seven and Eight from Addis Ketema and Woreda Six, Seven and Eight were from Akaki Kality sub cities.

The study sub-cities and woredas were selected purposively based on their high incidence of cholera cases in the 2019 outbreak in Addis Ababa. The simple population proportion formula was used to determine the sample size and a total of 206 samples consisting of drinking water (*n* = 97) and wastewater (*n* = 108) were considered for this study. Study households were selected following the systematic random sampling method. Sewage samples were collected from open sewage ditches close to selected households.

All drinking water samples were collected from point-of-use taps found in a total of 97 households from all study woredas each of the two study sub-cities. A volume of 250 mL of drinking water samples was aseptically collected using 500-ml sterile narrow-necked screw capped bottle. Similarly, 250 mLof wastewater samples were aseptically collected from 108 open ditch sewer sites found along innner roads in residential areas in both sub-cities. Collected samples were immediately transported to the laboratory in an icebox. Samples were processed within two hours of collection.

### Isolation of *V. cholerae* and *E. coli* O157:H7

A volume of 100 of a well-mixed sample was filtered through a 0.22-µm pore size polycarbonate filter (diameter 45 mm). The filter was placed in a 50 mL falcon tube containing 12 mL of sterile Phosphate-Buffered Saline (PBS) (OXOID) and vortexed vigorously for 5 min to suspend the attached bacteria into the saline solution.

To isolate *V. cholerae*, an aliquot of one mL of PBS suspension was added to an enrichment flask containing 25-ml Alkaline Peptone Water (APW) and incubated at 30-35^o^C for 18–24 h [34].

Surface growth from APW was streaked on pre-dried plates of Thiosulfate Citrate Bile salts Sucrose (TCBS) agar (OXOID) and incubated at 30-35^o^C for 18–24 h. All yellow colonies that fermented sucrose in TCBS agar were sub-cultured on Trypton Soy (TSY) Agar (OXOID). For cell morphology, wet mounts were examined microscopically at 40x using oil immersion. Colonies from TSY agar were also subjected to oxidase and string tests for *Vibrio cholearae.* Curved rods that had positive oxidase and string tests were presumptively considered as *V. cholerae* [34]. For serological confirmation, colonies were suspended in 0.5 mL physiological saline on a glass slide and gently mixed with a drop of antiserum for somatic O antigens by tilting the glass slide back and forth for one minute. Agglutination indicated a positive serological reaction. Serogroup O1 was similarly serotyped using type antisera.

To isolate *E. coli* O157:H7, 25 mL of the membrane filtered suspension was added to Phosphate Buffered Saline (PBS) and was incubated at 32 ºC for 18–24 h. The growth was directly streaked on pre-dried plates of Sorbitol MacConkey (SMAC) agar [35] and incubated at 32 ºC for 18–24 h. Non-sorbitol fermenting colorless colonies were further subjected for confirmation using *E. coli* O157:H7 Latex agglutination Test [36].

### Antimicrobial susceptibility testing for *V. cholerae* O1 and *E. coli* O157:H7 isolates

Antimicrobial susceptibility for *V. cholerae* and *E. coli* O157:H7 was determined by Kirby-Bauer disc diffusion method on Mueller-Hinton agar plates with commercially available antibiotic discs (OXOID) The 0.5 MacFarland was maintained using a 0.85% saline suspension of fresh colony from TSA agar and by measuring it using an OXOID MacFarland spectrometer.

*V. cholerae* Isolates were tested against 13 antimicrobial drugs consisting of amoxycillin (AML, 30 µg), azithromycin (AZM, 15 µg), ampicillin (AMP, 10 µg), peperacilin pazobactam (TZP, 110 µg), trimethoprim/sulphamethoxazole (STX, 25 µg), meropenem (MEM, 10 µg), cefoxitin (FOX, 10 µg), tetracycline (TE, 30 µg), ciprofloxacin (CIP, 5 µg), nalidixic acid (NA, 30 µg), ceftriaxone (CRO, 30 µg), ceftazidime (CAZ, 30 µg), ceftazidime/clavulanic acid (CAZ/CLA, 30/10 µg). After an overnight incubation of Mueller-Hinton agar, the inhibition zone of each antibiotic was measured [37].

*E. coli* O157:H7 isolates were also tested against 12 antimicrobial drugs consisting of ampicillin sulbactam (SAM 20 µg), amoxcillin clavulanic acid (AMC 30 µg), azithromycin (AZM, 15 µg), ampicillin (AMP, 10 µg), amikacin (AK, 30 µg), gentamicin (CN, 10 µg), trimethoprim/ sulphamethoxazole (SXT, 25 µg), imipenem (IPM, 10 µg), tetracycline (TET, 30 µg), ciprofloxacin (CIP, 5 µg), ceftriaxone (CRO, 30 µg), nitrofurantoin (F 300, µg). After an overnight incubation of Mueller-Hinton agar, the inhibition zone of each antibiotic was measured [38]. *E. coli* ATCC 25,922 strain was used as a positive control.

In both susceptibility tests a known positive control, and a blank disc were included. After an overnight incubation of Mueller-Hinton agar, the inhibition zone of each antibiotic was measured and recorded. For interpretation, the ‘Intermediate’ values were considered as ‘Sensitive’.

The multiple antibiotic resistance index (MARI) was calculated and interpreted according to Krumperman [39] using the formula: *a*/*b*, where ‘*a*’ represented the number of antibiotics to which an isolate was resistant, and *‘b*’ represented the total number of antibiotics tested.

### Determination of extended spectrum β-lactamase (ESBL) production and carbapenem resistance

Extended Spectrum Beta Lactamase (ESBL) producing *V. cholerae* and *E. coli* O157:H7 isolates was determined by using a double disk synergy test (DDST) [40]. Ceftazidime and Ceftazidime Clavulanic acid discs were employed and an inhibition zone diameter difference of ≥ 5 mm between the two drugs was considered as indication of ESBL production.

To detect Carbapenem Resistance among *V. cholerae* and *E. coli* O157: H7 resistance against Meropenem and Imipenem were checked, respectively. After an overnight incubation of Mueller-Hinton agar, the inhibition zone of both antibiotics was measured and recorded. The recorded size of inhibition zone was then changed into Sensitive (S), Intermediate (I) and Resistant (R) as per CLSI-M100. Resistance against the drugs were considered ar Carbapenem Resistance.

## Results and discussion

A total of 206 samples were collected from Addis Ketema (132) and Akaki/Kality (74) sub-cities. Of these, 97 (47.1%) were drinking water samples collected from point-of-use taps and 109 (52.9%) were wastewater samples from open sewers. *V. cholerae* O1 was isolated from 18 samples from Addis Ketema and 21 samples from Akaki/Kality sub-cities. Of the positive samples, 16 were drinking water samples and 23 were those from wastewater (Table 1). *V.cholerae* O1 isolates were dominated by Hikojima type (Table 1). Similar to the findings of this study, Ferdous et al. [7] detected *V. cholerae* in 10% of point-of-drinking water samples in a low-income urban community in Bangladesh. Similarly, the most frequently isolated *V. cholerae* O1 serotype from the sewage of Katmandu Valley, Nepal, was the Hikojima strain [12]. In a recent cholera outbreak in Addis Ababa, the responsible serotype belonged to the Ogawa type [18]. A review on cholera in Sub-Saharan Africa showed that Ogawa and Inaba serotypes were predominant [41]. *V. cholerae* O1 serotypes isolated from Kisumu county, Kenya were dominantly Inaba types followed by Ogawa [42]. According to Jubyda et al. [43], serotypes of *V. cholerae* O1 strains differed temporally in predominance in Bangladesh.

**Table 1:** *Vibrio cholerae* and *E. coli* O157:H7 isolated from wastewater and drinking water samples in the study sub-cities

Sub-city	Source (No. of samples)	Isolated species and serotypes	No of positive samples
Akaki/Kality	Wastewater (67)	*V. cholerae* O1 Hikojima *E. coli* O157:H7	14 12
	Drinking water (65)	*V. cholerae* O1 Hikojima *E. coli* O157:H7	7 9
Addis Ketema	Wastewater (42)	*V. cholerae* O1 Hikojima	8
		*V. cholerae* O1 Inaba	1
		*E. coli* O157:H7	7
	Drinking water (32)	*V. cholerae* O1 Hikojima *E. coli* O157:H7	9 1

A total of 28 strains of *E. coli* O157:H7 were isolated from the total samples of wastewater and drinking water in this study. The pathogen was encountered only in one samples of drinking water and seven samples of wastewater found in two woredas of Addis Ketema sub-city. Nine drinking water and 12 wastewater samples in Akaki/Kality sub-city, however, yielded *E. coli* O157:H7 (Table 1). Other authors also reported the isolation of *E. coli* O157:H7 from drinking water in Bangladesh [44] and in USA and Canada [45]. Schets et al. [22] isolated *E. coli* O157:H7 from 2.7% of samples in the Netherlands that otherwise met the drinking water standards. Momba et al. [44] reported that about 26% of their drinking water samples were positive for *E. coli* O157 in South Africa. Olsen et al. [21] reported that a large outbreak of *E. coli* O157:H7 infection which occurred in Wyoming, USA, was significantly associated with drinking municipal water. In fact, several outbreaks due to *E. coli* O157:H7 were strongly linked to the consumption of drinking water [46].

Wastewater would seep into the surrounding soil, eventually finding its way into drinking water through faulty water distribution lines. Interruptions of drinking water supply in Addis Ababa occur frequently. Resumption of supply would create negative pressures that would result in a suction effect inside the pipe, and pathogens in the surrounding would be sucked into the system through pipe leaks as observed by Collins and Boxall [9]. According to Ameya et al. [5]. , incorrect cross-connection with sewer lines, interconnection with toilets, pipe corrosion, and pipe breakage could lead to the infiltration of bacterial contaminants into water distribution lines. For this reason, Rashid et al. [47] recommended the use of chlorine tablets at point-of-use tabs to effectively inactivate *V. cholerae* from drinking water in households.

The contamination of drinking water by sewage was reported by Kwesiga et al. [14] in Western Uganda, which resulted in prolonged community-wide cholera outbreak. Shah et al. [48] found ten leakages in the drinking water pipelines of the affected areas during a cholera epidemic, caused by *V. cholerae* in Lalpur town, India. El-Leithy et al. [49] isolated *E. coli* O157:H7 from wastewater. Outbreaks of hemorrhagic colitis were linked to wastewater containing *E. coli* O157:H7 [50].

(Table 1)

Our *V. cholerae* O1 isolates exhibited different levels of resistance to the β-lactam antibiotics considered in this study: 100% resistance to two penicillins (Amoxicillin and Ampicillin), 51–82% resistance to the cephalosporins. About 44% of the isolates in this study were ESBL producers. Moreover, 23% were resistant to the only carbapenem, Meropenem, tested in this study and, possibly, could be carbapenemase producing strains. According to Goh [51], carbapenem-resistant *Vibrio* isolates have been identified in all continents and once carbapenem resistance is acquired among *Vibrio* isolates, the resistance genes may disseminate to other bacteria through mobile genetic elements and rapidly amplify the development of carbapenem resistance. Peperacillin/Tazobactam was the only effective β-lactam antibiotic against *V. cholerae* O1 in this study, because of its Tazobactam component, a β-lactamase inhibitor. (Table 2).

**Table 2:** Antimicrobial resistance of *V. cholerae* O1 isolated from drinking water and wastewater

Antibiotic Disc	Antibiotic class	Symbol	Concentration (µg)	No. of resistant isolates (%)
Amoxycillin	Penicillins	AML	30	39 (100)
Ampicillin	Penicillins	AMP	10	39 (100)
Azithromycin	Macrolides	AZM	15	18 (46.2)
Cefoxitin	Cephalosporins	FOX	30	21 (53.8)
Ceftazidime	Cephalosporins	CAZ	30	32 (82.1)
Ceftriaxone	Cephalosporins	CRO	30	20 (51.3)
Ciprofloxacin	Fluoroquinolones	CIP	5	9 (23.1)
Meropenem	Carbapenems	MEM	10	10 (25.6)
Nalidixic Acid	Quinolone	NA	30	32 (82.1)
Peperacillin/Tazobactam	Penicillins	TZP	100	2 (5.1)
Tetracycline	Tetracyclines	TE	30	25 (64.1)
Trimethoprim/Sulphamethoxazole	Sulfonamides	SXT	25	27 (69.2)
ESBL producers				17 (43.6)

Resistance to the Cephalosporins, Ciprofloxacin, Tetracycline and the Carbapenem (Meropenem) was much higher than that reported in other studies [28–30, 41].the *V. cholera* O1 strains in this study were, however, less resistant (69%) to Trimethprim Sulphamethoxazole than those of Garbern et al. [28] and Awuor et al. [42] which showed ≥ 99% resistance to the drug. Previous isolates of *V. cholera* O1 from Addis Ababa were sensitive to Tetracycline and Trimethprim/Sulphamethoxazole [18], whereas between 64% and 69% of the isolates in this study, respectively, were resistant to the two drugs.

(Table 2)

Our *E. coli* O157:H7 isolates showed varying levels of resistance to the nine antibiotic classes used in the study. About 71% were ESBL producing isolates (Table 3). Resistance to the β-lactam antibiotic Ampicillin was 96%. High degree of resistance of *E. coli* O157:H7 to Ampicillin was also reported by various authors [31, 32]. Resistance to Amoxycillin/Clavulanic acid and Ambicillin/Sulbactam was relatively lower (33% and 64%, respectively). Higher degree of resistance was, however, observed to Amoxicillin/Clavulanic Acid in other studies [25, 32], Both Aminoglycosides (Amikacin and Gentamicin) were very effective against the isolates in this study. Similar low resistance to Gentamicin was also reported by Hamid et al. [52] and Heydari et al. [53].

**Table 3:** Antimicrobial resistance of *E. coli* O157:H7 (*n* = 28) isolated from drinking water and wastewater

Antibiotic Disc	Antibiotic class	Symbol	Concentration (µg)	No. of resistant isolates (%)
Amikacin	Aminoglycosides	AK	30	0
Amoxcillin/Clavulanic Acid	Penicillins	AMC	30	9 (33.3)
Ampicillin	Penicillins	AMP	10	27 (96.4)
Ampicillin/Sulbactam	Penicillins	SAM	10	18 (64.3)
Azithromycin	Macrolides	AZM	15	7 (25)
Ceftriaxone	Cephalosporins	CRO	30	5 (17.9)
Ciprofloxacin	Fluoroquinolones	CIP	5	9 (33.3)
Gentamicin	Aminoglycosides	CN	10	1 (3.6)
Imipenem	Carbapenems	IPM	10	8 (28.6)
Nitrofurantoin	Nitrofuran	NIT	300	18 (64.3)
Trimethoprim-Sulphamethoxazole	Sulfonamides	SXT	25	16 (57.1)
Tetracycline	Tetracyclines	TE	30	18 (64.3)
ESBL producers				20 (71.4)

However, about 9% and 86% resistance to Gentamicin were reported Heydari et al. [52] and Haile et al. [25], respectively. Resistance to the Carbapenem (imipenem) and Cephalosporin (Ceftriaxone) is building up (29% and 18%, respectively) (Table 3) resulting in 28.6% of Carbapenem resistance. Yandag et al. [54] and Heyderi et al. [51] detected no resistance against Imipenem. Resistance to Ceftriaxone by isolates from water sources in Nigeria was 100% [32] whereas Haile et al. [25] reported no resistance to the drug. Unlike the isolates in this study, those of Tula et al. [32] showed complete resistance (100%) to Trimethoprim/sulphamethoxazole, Ampicillin, Amoxicillin/Clavulanic Acid and nalidixic acid.

### Multi-drug resistance (MDR) patterns of *V. cholerae* O1 and *E. coli* O157:H7 isolates

Our 39 *V. cholera* O1 isolates showed 37 different patterns of multiple antibiotic resistance against three to ten drugs. According to Jubyda et al. [43], *V. cholerae* strains differed in their antibiotic resistance pattern with a majority (97%) being multi-drug resistant to up to eleven of the eighteen antibiotics tested. This extreme drug resistant strain carried resistance-related genes that code for extended-spectrum *β*-lactamases [43]. The MAR index ranged from 0.3 to 0.8. Index values greater than 0.2 indicate that the origin of an isolate is a source where antibiotics are used to a great degree and/or in large amounts [39]. This would mean that, in the study areas considered in this study, antibiotics are accumulated in wastewater and, eventually in drinking water contaminated therewith. Igere et al. [55], determined the MDR of *V. cholerae* against 31 antibiotics and observed 33 MDR patterns consisting of nine to 23 drugs, with MAR index ranging from 0.03 to 0.5. Agboola et al. [56] isolated *V, cholerae* from hospital wastewater which showed multiple resistance against five to eight different antibiotic drugs with MAR index ranging from 0.4 to 0.6.

Although we noted 37 different patterns of multiple antibiotic resistance in *V, cholerae* O1 isolates, there were few repeating segments within the patterns. The most frequently appearing segments were AML/AMP/AZM (48.7%); CRO/CAZ/NA (38.5%); AML/AMP/AZM/SXT (30.7%); CAZ/CRO/NA/TE (23.1%); and AML/AMP/AZM/FOX/SXT (23.1%). The single *V.cholerae* O1 isolate from drinking water collected from Addis Ketema sub-city was not multiple drug resistant. Those from drinking water collected from Akaki/Kality sub-city, however, showed a higher magnitude of multiple drug resistance, mostly resistance to six to nine drugs (Table 4). This indicates that drinking water in distribution lines in Akaki/Kality sub-city is more prone to contamination from environmental sources.

**Table 4:** Multiple drug resistance patterns of *V. cholerae* O1 and *E. coli* O157:H7 isolated from drinking water

Isolate	Sub-city	Sample ID^1^	MDR pattern^2^	MAR index
*V. cholerae* O1	Addis Ketema	ADW3DW22	CAZ/NA	0.2
		ADW3DW7	AML/ AMP/ CRO	0.3
		ADW3DW12	AML/ AMP/ FOX/ NA/SXT/TE	0.5
		ADW7DW16	AML/ AMP/ CAZ/ NA/SXT/ TE	0.5
		ADW7DW17	AML/ AMP/CAZ	0.3
		ADW7DW10	AML/ AMP/ CAZ/ CRO/ FOX/SXT	0.5
		ADW8DW9	AML/ AMP/ CAZ/CIP/ FOX/ MEM/SXT	0.6
		ADW8DW19	AML/ AMP/CAZ/CIP/TE	0.4
	Akaki/ Kality	AKW6DW1	AML/ AMP/AZM/ CAZ/ CRO/ FOX/ SXT/ TE	0.7
		AKW6DW7	AML/ AMP/ CRO/ FOX/SXT/TE	0.5
		AKW6DW8	AML/ AMP/ SXT/ CAZ/CRO/FOX/NA	0.6
		AKW6DW10	AML/ AMP/ CIP/MEM/ NA/ SXT	0.5
		AKW7DW1	AML/ AMP/AZM/ CAZ/CRO/ FOX/ NA/SXT/TE	0.8
		AKW8DW6	AML/ AMP/ FOX/MEM/ NA/SXT	0.5
		AKW8DW3	AML/ AMP/ FOX/NA/TZP	0.4
*E. coli* O157:H7	Addis Ketema	ADW8DW8	AMP/ IMP	
	Akaki/ Kality	AKW6DW6	AMC/AMP/ CRO/ MP/NIT/SAM	0.5
		AKW6DW8	AMC/AMP/ SAM/ IMP/ CIP/NIT/TE/ CRO	0.7
		AKW6DW4	AMC/AMP/SAM	0.3
		AKW8DW3	AMP/SAM/ SXT	0.3
		AKW8DW9	AMP/SAM/SXT	0.3
		AKW8DW8	AMC/AMP/SAM	0.3
		AKW8DW5	AMP/SAM/ SXT	0.3
		AKW8DW4	AMC/AMP/NIT/SAM/SXT	0.4

^1^Sample ID; AD, Addis Ketema sub-city; AK, Akaki/Kality sub-city; W, Woreda; DW, drinking water; WW, wastewater

^2^Complete MDR pattern of all isolates is given in Annex I

About 89% of the *E. coli* O157:H7 isolates from wastewater and drinking water showed MDR against three to eight antibiotic drugs. Resistance to up to 12 drugs was reported by Tula et al. [32]. Lower proportions (31–68%) of MDR *E. coli* O157:H7 isolates were reported by various authors [24, 25, 32, 50]. Four each of the isolates in this study were resistant to four and five drugs. Most patterns (89%) were different from one another. Five were resistant to three drugs, and the most frequent pattern was AMP/SAM/SXT.

The presence of *V. cholerae* O1 and *E. coli* O157:H7 in drinking water samples (Table 4) exposes residents of the study areas to recurring disease that could be fatal, particularly to vulnerable members of households. Moreover, multiple antibiotic-resistant pathogens, when introduced to the human gut, would result in further conjugal transfer of plasmids, that carry antibiotic resistance genes, to the normal gut microbiota. The gut would, thus, be a permanent source of MDR microorganisms to the individual and the environment [57]. According to Ceccarelli et al. [58], enteric pathogens release β-lactam resistant genes to the environment and *V. cholerae* has the ability to acquire new genetic information therefrom through horizontal gene transfer mechanisms.

An *E. coli* O157:H7 isolate from drinking water samples was multiple drug resistant to eight drugs. More than half were resistant only to three drugs. AMC/AMP/SAM and AMP/SAM/SXT appeared more frequently than the other patterns. Multiple antibiotic resistance index of the *E. coli* O157:H7 strains isolated from wastewater and drinking water ranged from 0.3 to 0.8. The MAR index of isolates from drinking water ranged between 0.2 and 0.7 (Table 4). Different multiple antibiotic resistance indices were reported for *E. coli* O157:H7: 0.2 to 0.7 [59] and 0.6 to 1.0 [60].

Similarly, the *V. cholerae* O1 isolates from drinking water samples collected from Addis Ketema sub-city manifested MDR against three to seven antibiotic drugs (MARI, 0.2-0.05). However, those isolates from samples collected from Akaki/Kality sub-city showed MDR against five to nine drugs (MARI, 0.4, 0.8) (Table 4). This indicates that drinking water in Akaki/Kality sub-city is more contaminated with MDR *V. cholerae* O1 strains than that in Addis Ketema sub-city.

The MAR pattern as well as the indices of all *V. cholerae* O1 and *E. coli* O157:H7 isolates, including those from wastewater samples, are given in annex 1 and 2.

## Conclusion

Previous cholera outbreaks were reported from all sub-cities at different times. This study addressed only a few woredas in only two sub-cities. It has, however, shown that open ditches for wastewater conveyance along innner roads in residence areas and underground faulty municipal water distribution lines could be major sources for *V. cholerae* O1 and *E. coli* O157:H7 infections to surrounding households. The isolation of both pathogens, particularly from point-of-use drinking water taps, makes the quality of municipal drinking water in the city questionable. Thus, consumers may be advised to treat drinking water immediately after collection from point-of-use taps by boiling or adding other treatment chemicals before consumption or storage. On the other hand, it is crucial to manage open sewer ditches by communities and occasionally check the integrity of drinking water distribution lines by the responsible government bodies to achieve the ‘Multi-sectorial Cholera Elimination Plan, Ethiopia 2021–2028’, which targets to end cholera by 2030 as part of the Global Roadmap [61].

### Electronic supplementary material

Below is the link to the electronic supplementary material.

Supplementary Material 1

Supplementary Material 2

## Data Availability

Data is provided within the manuscript or supplementary information files.
