# Correction: *Vibrio cholerae* O1 and *Escherichia coli* O157:H7 from drinking water and wastewater in Addis Ababa, Ethiopia

**DOI:** 10.1186/s12866-024-03405-2

**Published:** 2024-07-05

**Authors:** Helina Mogessie, Mengistu Legesse, Aklilu Feleke Haile, Tilahun Teklehaymanot, Haile Alemayehu, Rajiha Abubeker, Mogessie Ashenafi

**Affiliations:** 1 Microbiology Research Unit, Aklilu Lemma Institute of Pathobiology, Addis Ababa University, Addis Ababa, Ethiopia; 2 Ethiopian Public Health Institute, Bacteriology Directorate, Addis Ababa, Ethiopia; 3 Center for Food Security Studies, College of Development Studies, Addis Ababa University, Addis Ababa, Ethiopia

**Correction:** ***BMC Microbiol*** **24, 219 (2024)**

10.1186/s12866-024-03302-8

Following the publication of the original article [1], the authors would like to change the third co-author’s name, which is “Aklilu Feleke Hailu”, with “Aklilu Feleke Haile”.
