# Supplementary material for: Vibrio cholerae O1 and Escherichia coli O157:H7 from drinking water and wastewater in Addis Ababa, Ethiopia
Source: BMC Microbiol. 2024 Jun 20;24:219. doi: 10.1186/s12866-024-03302-8 (PMC11188251; doi:10.1186/s12866-024-03302-8)
Supplement: Supplementary file 1 — Supplementary Material 1 [file 12866_2024_3302_MOESM1_ESM.docx]

ANNEX 1

Annex 1. Drug resistance patterns of *V. cholerae* O1 isolates collected from drinking water and wastewater.

| Sample ID* | Resistant  to | Resistance pattern | MAR  index |
| --- | --- | --- | --- |
| AKW6DW1 | Ten | AML/ AZM/ AMP/ SXT/ FOX/ TE/ CIP/NA/CRO/ CAZ |  |
| AKW8DW6 |  | AML/ AZM/ AMP/ SXT/ FOX/ TE/ CIP/NA/CRO/ CAZ |  |
| AKW8WW14 |  | AML/ AZM/ AMP/ TZP/MEM/FOX/ TE/ CIP/NA/ CAZ | 0.8 |
| AKW7WW4 |  | AML/ AZM/ AMP/ SXT/ FOX/ TE/ CIP/NA/CRO/ CAZ |  |
| AKW7WW8 |  | AML/ AZM/ AMP/ TZP/ SXT/ FOX/ TE/ NA/CRO/ CAZ |  |
| AKW7WW6 |  | AML/ AMP/ SXT/MEM/ FOX/ TE/ CIP/NA/CRO/ CAZ |  |
| ADW7WW19 | Nine | AML/ AZM/ AMP/ SXT/ FOX/ TE/NA/ CRO/ CAZ |  |
| ADW7WW7 |  | AML/ AZM/ AMP/ SXT/ FOX/ TE/ NA/CRO/ CAZ | 0.8 |
| ADW8WW19 |  | AML/ AZM/ AMP/ SXT/MEM/ FOX/TE/ NA/CAZ |  |
| AKW7WW10 |  | AML/ AZM/ AMP/ SXT/MEM/ FOX/ CIP/NA/CAZ |  |
| ADW8DW9 | Eight | AML/ AZM/ AMP/ SXT/ FOX/ TE/NA/ CAZ |  |
| AKW6DW8 |  | AML/ AZM/ AMP/ SXT/TE/ NA/CRO/ CAZ |  |
| AKW7DW1 |  | AML/ SXT/MEM/ FOX/ TE/NA/CRO/ CAZ |  |
| ADW7DW16 |  | AML/ AZM/ AMP/ SXT/ FOX/ NA/CRO/ CAZ | 0.7 |
| ADW7DW17 |  | AML/ AMP/ SXT/ FOX/ TE/ NA/CRO/ CAZ |  |
| ADW3DW12 |  | AML/ AZM/ AMP/ SXT/ FOX/ TE/ NA/ CAZ |  |
| AKW6WW10 |  | AML/ AZM/ AMP/ SXT/ FOX/ TE/ CRO/ CAZ/ |  |
| AKW6DW7 |  | AML/ AMP/ SXT/MEM/ TE/ NA/ CAZ |  |
| ADW7DW10 | Seven | AML/ AMP/ SXT/ FOX/NA/CRO/ CAZ |  |
| ADW3DW22 |  | AML/ AMP/ SXT/MEM/ TE/ NA/ CAZ | 0.6 |
| ADW3DW7 |  | AML/ AMP/ SXT/ TE/ NA/CRO/ CAZ |  |
| ADW3WW23 |  | AML/ AZM/ AMP/ TE/ NA/CRO/ CAZ |  |
| ADW3WW9 |  | AML/ AMP/ SXT/MEM/ FOX/ CIP/ CAZ |  |
| AKW8DW3 | Six | AML/ AZM/ AMP/ TE/ NA/ CAZ |  |
| ADW7WW17 |  | AML/ AZM/ AMP/NA/CRO/CAZ |  |
| ADW7WW10 |  | AML/ AMP/SXT/MEM/FOX/NA |  |
| AKW8WW8 |  | AML/ AMP/ SXT/ TE/ NA/ CAZ | 0.5 |
| AKW8WW10 |  | AML/ AMP/ SXT/ FOX/ TE/ NA |  |
| AKW8WW7 |  | AML/ AMP/ SXT/ FOX/ CRO/ CAZ |  |
| AKW8WW1 |  | AML/ AMP/ SXT/ FOX/ TE/ CRO |  |
| ADW7WW8 |  | AML/ AMP/ SXT/MEM/ CIP/NA/ |  |
| AKW8WW5 | Five | AML/ AZM/ AMP/TE/CAZ |  |
| AKW8WW9 |  | AMP/ TE/ NA/CRO/ CAZ | 0.4 |
| AKW8WW2 |  | AML/ AMP/ TZP/ FOX/ NA |  |
| ADW8WW23 |  | AML/ AMP/ TE/ CIP/ CAZ |  |
| AKW6DW10 | Three | AML/ AMP/ CRO |  |
| AKW6WW9 |  | AML/ AMP/CAZ | 0.3 |
| AKW6WW11 |  | AML/ AMP/ NA |  |

*Sample ID; AD, Addis Ketema sub-city; AK, Akaki/Kality sub-city; W, Woreda; DW, drinking water; WW, wastewater.
