# Supplementary material for: Vibrio cholerae O1 and Escherichia coli O157:H7 from drinking water and wastewater in Addis Ababa, Ethiopia
Source: BMC Microbiol. 2024 Jun 20;24:219. doi: 10.1186/s12866-024-03302-8 (PMC11188251; doi:10.1186/s12866-024-03302-8)
Supplement: Supplementary file 2 — Supplementary Material 2 [file 12866_2024_3302_MOESM2_ESM.docx]

ANNEX 2

Annex 2. Drug resistance patterns of *E. coli* O157:H7 isolates collected from drinking water and wastewater.

| Sample ID* | Resistant  to | | Resistance pattern | MAR  index |
| --- | --- | --- | --- | --- |
| AKW7WW12 | Nine | AMP/AMC/SAM/ IMP/ CN**/** NIT/TE/ SXT/CRO | | 0.8 |
| AKW6DW8 | Eight | AMP/AMC/SAM/ IMP/ CIP/ NIT/TE/ CRO | | 0.7 |
| AKW6WW6 |  | AMP/AMC/ CIP/ NIT/TE/ SXT/AZM/CRO | |  |
| AKW8WW11 | Seven | AMP/ AMC/ CIP/ NIT/SXT/AZM/CRO | |  |
| ADW8WW21 |  | AMP/ AMC/SAM/ CIP/ NIT/TE/AZM | | 0.6 |
| ADW3WW11 |  | AMP/ AMC/SAM/ CIP/ NIT/TE/ SXT | |  |
| AKW6DW6 | Six | AMP/AMC/SAM/ IMP/NIT/CRO | |  |
| AKW7WW13 |  | AMP/ SAM/ IMP/ NIT/TE/ SXT | | 0.5 |
| ADW8WW20 |  | AMP/ AMC/SAM/ NIT/TE/ SXT | |  |
| ADW8WW13 |  | AMP/ SAM/ CIP/ NIT/TE/SXT | |  |
| AKW8DW4 | Five | AMP/AMC/SAM/ NIT/ SXT | |  |
| AKW8WW14 |  | AMP/ AMC/ IMP/SXT/AZM | | 0.4 |
| ADW8WW17 |  | AMP/ CIP/ NIT/TE/ AZM | |  |
| ADW8WW18 |  | AMP/AMC/ NIT/TE/SXT | |  |
| AKW6 WW7 | Four | IMP/ CIP/ NIT/TE | |  |
| AKW6WW5 |  | AMP/ CIP/ NIT/TE | |  |
| AKW6WW12 |  | AMP/ NIT/TE/SXT | | 0.3 |
| AKW8WW4 |  | AMP/ NIT/ AK/SXT | |  |
| ADW8WW15 |  | AMP/AMC/ NIT/TE | |  |
| AKW8DW3 | Three | AMP/ SAM/ SXT | |  |
| AKW6DW4 |  | AMP/ AMC/SAM | |  |
| AKW8DW9 |  | AMP/SAM/SXT | | 0.3 |
| AKW8DW8 |  | AMP/ AMC/SAM | |  |
| AKW8DW5 |  | AMP/ SAM/ SXT | |  |
| AKW7WW3 |  | AMP/ IMP/ TE | |  |
| AKW8WW12 | Two | AMP/ SAM | |  |
| AKW8WW10 |  | AMP/ TE | |  |
| ADW8DW8 |  | AMP/ IMP | |  |

*Sample ID; AD, Addis Ketema sub-city; AK, Akaki/Kality sub-city; W, Woreda; DW, drinking water; WW, wastewater.
